# Bravery

**Published:** 1879-11

**Authors:** 


### BRAVERY.

EVERY man thinks himself brave until the battle. We have long been under the impression that we feared nothing and nobody, in spite of some dreamy intimations now and then. It is generally supposed, that, as a man acts in his dreams, he would act under the same circumstances if awake. By this rule, we are neither one thing nor the other, but rather, if anything, the other, as to bravery. We have frequently dreamed of battles, and invariably there was a contest within, and a question—a dilemma with three horns, and all sharp. We wanted to run away, but was afraid of the disgrace; we wanted to fight, for the credit of being considered brave, but were afraid of the bullet; and we wanted to be *statu quo*, and thus be on the safe side both ways! Once we were fairly driven "to the scratch," most sorely against our will, and, at the very first crack, we "hollered," and waked up.

